# Provision of social support and mental health in U.S. military veterans

**DOI:** 10.1038/s44184-022-00004-9

**Published:** 2022-06-29

**Authors:** Peter J. Na, Jack Tsai, Steven M. Southwick, Robert H. Pietrzak

**Affiliations:** 1 VA Connecticut Healthcare System, West Haven, CT USA; 2 Department of Psychiatry, Yale School of Medicine, New Haven, CT USA; 3 U.S. Department of Veterans Affairs National Center on Homelessness Among Veterans, Tampa, FL USA; 4 School of Public Health, University of Texas Health Science Center at Houston, San Antonio Campus, San Antonio, TX USA; 5 National Center for PTSD, VA Connecticut Healthcare System, West Haven, CT USA; 6 Department of Social and Behavioral Sciences, Yale School of Public Health, New Haven, CT USA

**Keywords:** Medical research, Risk factors

## Abstract

While social support has been linked to better health, most research has focused on the receipt of social support. In this study, we evaluated associations between provided support and mental health in a nationally representative cohort of 4069 US veterans. The majority (60–72%) of veterans reported providing support on a consistent basis. Veterans who scored higher on certain aspects of personality (i.e., agreeableness, conscientiousness, and extraversion) and received greater support were more likely to provide support. Further, each standard deviation increase in provided support was independently associated with 22–32% reduced odds of internalizing psychiatric disorders and suicidal ideation, and veterans who scored higher on both provided and received support had 3.5- to 14-fold lower odds of these outcomes relative to those with high received support but low provided support. Results suggest that interventions to promote the provision of support may help mitigate risk for adverse mental health outcomes in veterans.

## Introduction

*If we focus too much on ourselves, we’ll not be happy, whereas to concern ourselves with the well-being of others is the gateway to great joy*. - Dalai Lama^1^.

Since the seminal article by House and colleagues^2^ published more than three decades ago, social support has been thought to improve health and lengthen lives^3^. Social support is defined as the perception or experience of being loved and cared for by others^4^. Previous research identified the impact of lack of social support as a strong risk factor for all-cause mortality rates from various morbidities^5,6^, including cardiovascular disease^7^, cancer, and infectious diseases^6^. Further, accumulating research has shown positive associations between social support and measures of well-being, including cognitive functioning^8^.

In the mental health literature, a recent systematic review of 34 studies found substantial evidence that poorer social support is prospectively linked to worse symptom severity, recovery outcomes and social functioning in individuals with depression^9^. Although less robust, preliminary evidence suggests that poor social support is associated with negative outcomes, such as greater severity of depressive symptoms and functional impairment, and longer time to recovery in individuals with other mental disorders, such as schizophrenia, bipolar disorder and anxiety disorders^9^.

To date, the majority of research on social support and health outcomes has focused on *receipt* of social support, and its impact on mental and physical health outcomes^6,10,11^. In contrast, substantially less attention has been given in examining the association between the *provision* of social support to others and health outcomes. In one of the first studies to examine the relationship between received and provided social support and mortality, the association between receiving social support and mortality was found to be moderated by the provision of social support^12^. Specifically, while receiving social support reduced the risk of mortality, this effect was nullified after adjusting for the provision of social support^12^. More recent work further suggests that the provision of social support may also be associated with longevity and positive health outcomes^11–13^.

Extant research on the association between the provision of social support and health outcomes is limited in several ways. First, the majority of published studies have focused on the impact of providing social support and physical health outcomes, such as mortality rate. Previously, there have been studies that examined the association between volunteering/civic engagement in veterans and depression^14,15^. However, to our knowledge, no known study has examined the associations between the provision of social support and a comprehensive range of adverse mental health outcomes such as major depressive disorder (MDD), posttraumatic stress disorder (PTSD), generalized anxiety disorder (GAD) and suicidal ideation (SI). Second, existing studies have not taken into consideration the potential interaction effects between the provision of social support and other aspects of support, such as structural support (i.e., number of supportive friends or family members) and received social support (i.e., perceived support from others) in relation to mental health outcomes. Third, previous literature has focused on the general population, and less is known about the association between providing social support and mental health outcomes in higher-risk populations, such as US military veterans. Characterizing the role of provided and other aspects of support in relation to prevalent psychiatric outcomes may help inform targeted clinical and policy interventions to improve mental health outcomes in such populations.

U.S. military veterans are an ideal population in which to examine this topic given national concern about their well-being; ongoing efforts to help veterans with re-integration into civilian life; and the abundance of research on peer support and received social support among veterans, but the relative paucity of research on provided support. For instance, we previously found that among veterans returning from deployment to Iraq and Afghanistan, greater perceptions of received social support mediated the relation between PTSD and social functioning^16^.

Using data from the 2019–2020 National Health and Resilience in Veterans Study (NHRVS), which surveyed a nationally representative sample of more than 4000 US veterans, we evaluated the prevalence of different types of provided support and sociodemographic, military, health, and psychosocial characteristics associated with greater provision of social support. The primary analyses of this study assessed the independent and interactive associations between provision of social support and other aspects of support in relation to current internalizing mental disorders (i.e., MDD, GAD, PTSD) and SI.

## Methods

### Participants

Data were analyzed from the NHRVS, a nationally representative survey of US military veterans. A total of 4069 veterans completed a 50-min online survey (median completion date: 11/21/2019, survey period: 11/18/2019-03/08/2020). A total of 7860 veterans were invited and 4069 (51.8%) completed the survey. Details of the study, including the recruitment protocol, has been described previously^17^. Briefly, the NHRVS sample was drawn from KnowledgePanel^®^, a survey research panel of more than 50,000 US households maintained by Ipsos, a survey research firm. To ensure generalizability of the results to the US veteran population, poststratification weights were computed based on the demographic distribution of veterans in the Veterans Supplement of the US Census Current Population Survey. The study protocol was approved by the Human Subjects Subcommittee of the VA Connecticut Healthcare System, and all participants provided informed consent.

### Sociodemographic characteristics

Age, gender (male vs. female), race (White, non-Hispanic vs. other), education (college graduate or higher vs. some college or lower), marital status (married/living with partner vs. unmarried/unpartnered), annual household income ($60,000 or more vs. less than $60,000), employment status (retired vs. working), combat veteran status, military enrollment status (enlisted/commissioned vs. drafted), and years in military service (10 years or more vs. less than 10 years) were assessed.

### Positive effect of military on life

Rating on the item: How has being in the military affected your life? Score range: 1–7 (1 = Strong negative effect; 7 = Strong positive effect).

### Number of medical conditions

Sum of number of medical conditions endorsed in response to question: “Has a doctor or healthcare professional ever told you that you have any of the following medical conditions?” (e.g., arthritis, cancer, diabetes, heart disease, asthma, kidney disease). Range: 0–24 conditions.

### Adverse childhood experiences

Score on Adverse Childhood Experiences Questionnaire^18^, which provides a count of adverse childhood experiences (range: 0–10).

### Potentially traumatic events

Count of direct (i.e., happened to me) and indirect (i.e., witnessed, learned about, and part of job) potentially traumatic events on the Life Events Checklist for DSM-5^19^.

### Lifetime psychiatric diagnoses

Lifetime diagnoses of MDD, alcohol, and drug use disorder were assessed using a modified self-report version of the Mini International Neuropsychiatric Interview for DSM-5^20^. Lifetime PTSD was assessed using a lifetime version of the PTSD Checklist for DSM-5; a score ≥ 33 was indicative of a positive screen for PTSD^21^.

### Personality

The Ten-Item Personality Inventory (TIPI) was used to measure the “Big Five” personality traits of emotional stability (anxious vs. confident and calm), extraversion (outgoing vs. reserved), openness to experiences (imaginative and inventive vs. cautious/routine-like), agreeableness (friendly and cooperative vs. detached), and conscientiousness (efficient and organized vs. careless). Respondents rate the ten items on a seven-point Likert scale (1 = disagree strongly to 7 = agree strongly). Items are paired into five sets of two items.

### Provision of social support

Level of provided social support was assessed using a modified 5-item version of the Medical Outcomes Study Social Support Scale^22,23^. Participants were asked “How often do you provide each of the following kinds of support to others who need it?” on a five-point scale from (1) *none of the time* to (5) *all the time*. Examples of items were: “I am someone that others could confide in or talk to about their problems” and “I am someone that others could get together with for relaxation.” Scores range from 5 to 25, with higher scores indicative of greater social support (Cronbach’s α = 0.88).

### Structural social support

Structural social support was assessed with the question: “About how many close friends and relatives do you have – people you feel at ease with and can talk to about what is on your mind?” This was scored as a count, ranging from 0 to 90.

### Days visit family/friends per week

Frequency of social engagement was associated using the following questions: “How many days per week do you typically engage in the following activities?” Visiting family; Visiting friends (response options: 0–7).

### Receipt of social support

Level of received social support was measured using a 5-item version of the Medical Outcomes Study Social Support Scale^22,23^. Participants were asked “How often each of the following kinds of support are available to you if you need it?” on a five-point scale from (1) *none of the time* to (5) *all the time*. Examples of items were: “Someone to confide in or talk about your problems” and “Someone to get together with for relaxation”. Scores range from 5 to 25, with higher scores indicative of greater social support (Cronbach’s α = 0.89). This measure primarily assesses perceptions of received social support from others and does not assess structural social support (i.e., number of supportive others).

### Current psychiatric symptoms

MDD symptoms were assessed using the two depressive symptoms of the PHQ-4 (Cronbach’s α = 0.87); a score ≥ 3 was indicative of a positive screen for MDD^24^. PTSD symptoms were assessed using the PTSD Checklist for DSM-5 (Cronbach’s α = 0.96); a score ≥ 33 was indicative of a positive screen for PTSD^21^. GAD symptoms were assessed using the two generalized anxiety items of the PHQ-4 (Cronbach’s α = 0.86); a score ≥ 3 was indicative of a positive screen for GAD^24^.

#### Alcohol use disorder (AUD)

Probable AUD were assessed using the Alcohol Use Disorders Identification Test (AUDIT), a validated measure used to screen for AUD. The AUDIT consists of 10 questions that assess the severity of alcohol consumption and consequences and yield a total score ranging from 0 to 40. Higher scores indicate more severe problematic alcohol use. A cut-off score of 8 or higher was considered as indicative of probable AUD^25^.

#### Drug use disorder (DUD)

How many days in the past year have you used non-prescription drugs? A response of ≥ 7 days on this question is indicative of a positive screen for DUD; if the response to this question is 6 or fewer days, a response of ≥ 2 days to the question “How many days in the past 12 months have you used drugs more than you meant to?” is indicative of a positive screen for drug use disorder.

### Suicidal ideation

SI was assessed using two items adapted from the PHQ-9 Item 9^26^, which asked participants to report suicidal thoughts during the prior two weeks. A positive screen was indicated by a response of “several days,” “more than half the days,” or “nearly every day” to at least one of the following questions: “How often have you been bothered by thoughts that you might be better off dead?” and “How often have you been bothered by thoughts of hurting yourself in some way?”

### Statistical analysis

Item-level missing data (<5%), which were missing completely at random (MCAR) as per Little’s MCAR test (all p’s > 0.50), were imputed using chained equations. Data analyses proceeded in five steps. First, descriptive statistics were computed to summarize sample characteristics and prevalence of each aspect of provided support (i.e., None/A Little of the Time; Some of the Time; Most/All of the Time). Second, correlations (Pearson for continuous independent variables; point-biserial for dichotomous independent variables) were computed between provided support scores, and sociodemographic, military, and health and psychosocial characteristics. Third, a multiple regression analysis was conducted to identify factors that were independently associated with provided support scores; variables that were significantly associated with provided support scores at the p < 0.15 level in bivariate analyses were included in this analysis. Fourth, we conducted a series of multivariable hierarchical binary logistic regression analyses to examine the relation between provided, structural, and received support (scores were standardized for ease of interpretation), and dependent variables. Main effects were evaluated in Step 1 of these models; and then interaction terms of provided x structural, and provided x received support were added in Step 2 of these models. To correct for multiple testing (i.e., 4 primary dependent variables), alpha was set to 0.0125 (0.05/4 dependent variables) for these analyses. Fifth, post-hoc analyses were conducted to identify specific aspects of provided support associated with these variables; due to the exploratory nature of these analyses, alpha was set to 0.05.

## Results

### Study results

The average age of the sample was 62.2 years (standard deviation [SD] = 15.7: range 22–99); the majority of whom were male (90.2%) and White, non-Hispanic (78.1%), and 35.0% were combat veterans. The mean score on the provided support measure was 19.0 (SD = 4.3, range = 5–25).

**Fig. 1:**
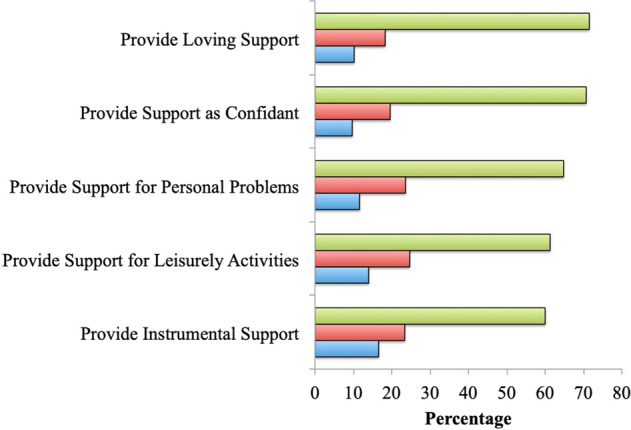
Prevalence of frequency of provided social support among U.S. military veterans. Green Bar – Most/All the Time, Red Bar – Some of the Time, Blue Bar – None/Little of the Time.

Fig. 1 shows the prevalence of different aspects of provided support. The majority of veterans endorsed providing each type of support most or all of the time, the most prevalent of which were providing loving (71.5%) and confiding (70.7%) support, and support for personal problems (64.8%).

Table 1 shows sample characteristics and results of bivariate and multivariable analyses of correlates of provided support. Multivariable analysis revealed that veterans who were married/partnered, perceived a positive effect of the military on their lives, experienced more direct and indirect potentially traumatic events, and who scored higher on measures of the ‘Big 5’ personality indicators, structural support, and received support, and reported greater frequency of visiting family were positively associated with provided support. Male gender and greater number of medical conditions were negatively associated with this measure.

**Table 1:** Sample characteristics and correlates of provision of social support in U.S. veterans.

	Weighted mean (SD) or n (weighted %)	Correlation	Multivariable regression analysis (*R*^2^ = 0.48)			
		*r*	β	*B*	SE	*p*
*Sociodemographic characteristics*						
Age	62.2 (15.7)	0.02	–	–	–	
Male gender	3564 (90.2%)	−0.04*	−0.05	−0.83	0.17	<0.001
White, non-Hispanic race/ethnicity	3318 (78.1%)	0.02	–	–	–	–
College graduate or higher education	1827 (32.7%)	0.06***	−0.01	−0.11	0.11	0.36
Married or partnered	2885 (72.4%)	0.14***	0.03	−0.23	0.12	0.14
Retired	2225 (44.3%)	−0.01	–	–	–	
Household income $60 K or higher	2357 (58.5%)	0.14***	0.02	0.18	0.11	0.10
*Military characteristics*						
Enlisted/Commissioned vs. Drafted	3583 (89.1%)	−0.01	–	–	–	–
Combat veteran	1353 (35.0%)	0.01	–	–	–	–
10+ years in military	1476 (36.4%)	0.03	–	–	–	–
Positive effect of military on life	2.0 (1.4)	0.24***	0.08	0.23	0.04	<0.001
*Health characteristics*						
Number of medical conditions	2.9 (2.2)	−0.08***	−0.03	−0.06	0.02	0.002
Adverse childhood experiences	1.5 (2.0)	−0.12***	0.01	0.03	0.03	0.39
Direct PTEs	3.2 (2.5)	0.01	0.05	0.09	0.02	<0.001
Indirect PTEs	5.7 (7.1)	0.04**	0.03	0.02	0.01	0.002
Lifetime MDD and/or PTSD	833 (22.4%)	−0.16***	0.02	0.28	0.14	0.052
Lifetime AUD and/or DUD	1673 (42.8%)	−0.08***	0.02	0.21	0.11	0.061
*Personality*						
Extraversion	3.8 (1.5)	0.30***	0.07	0.21	0.04	<0.001
Agreeableness	5.0 (1.2)	0.32***	0.12	0.44	0.05	<0.001
Conscientiousness	5.7 (1.2)	0.36***	0.12	0.43	0.05	<0.001
Emotional stability	5.2 (1.4)	0.36***	0.03	0.11	0.05	0.027
Openness to experiences	4.8 (1.2)	0.29***	0.10	0.34	0.05	<0.001
*Social variables*						
Structural social support	8.1 (10.9)	0.24***	0.03	0.01	0.005	0.014
Days visit family per week	1.7 (1.9)	0.15***	0.03	0.09	0.03	0.002
Days visit friends per week	1.7 (1.6)	0.19***	0.00	0.02	0.03	0.26
Received social support	19.0 (4.3)	0.61***	0.49	0.41	0.01	<0.001

*PTEs* potentially traumatic events, *MDD* major depressive disorder, *PTSD* posttraumatic stress disorder, *AUD* alcohol use disorder, *DUD* drug use disorder. β standardized coefficient, *B* unstandardized coefficient, *SE* standard error.

Significant association in correlation analysis: *p < 0.05, **p < 0.01, ***p < 0.001.

Exploratory post-hoc analyses of individual aspects of provided support revealed that higher scores on being a confidant to others (odds ratio [OR] = 0.79, 95% confidence interval [CI] = 0.70–0.89) was associated with lower odds of MDD; being someone others can rely on for relaxing activities with lower odds of GAD (OR = 0.84, 95%CI = 0.71–0.99); being someone who can help others with daily chores with lower odds of suicidal intent (OR = 0.55, 95%CI = 0.42–0.73); being someone others can turn to for help with personal problems with lower odds of GAD (OR = 0.79, 95%CI = 0.67–0.92); and being someone others can love with lower odds of PTSD (OR = 0.80, 95%CI = 0.69–0.92) and SI (OR = 0.82, 95%CI = 0.73–0.92).

In the full sample, a total of 292 (8.7%), 232 (7.8%), and 219 (6.6%) veterans screened positive for current MDD, GAD, PTSD, respectively, and 387 (11.7%) endorsed past-year SI. Table 2 shows the results of multivariable analyses of the relation between provided, structural, and received support, and these psychiatric outcomes. Results of the analysis of the Step 1 analysis of main effects revealed that greater provided support was negatively associated at the corrected *p* value < 0.0125 with all variables except PTSD; structural support with MDD and SI; and received support with all variables. Significant interactions between provided and received support were observed for MDD, GAD, and SI.

**Table 2:** Associations between provided, structural, and received social support, and dependent variables.

	Major Depressive Disorder			Generalized Anxiety Disorder			Posttraumatic Stress Disorder			Suicidal Ideation		
	B (SE)	p	OR (95%CI)	B (SE)	p	OR (95%CI)	B (SE)	p	OR (95%CI)	B (SE)	p	OR (95%CI)
Provided support	−0.25 (0.07)	<0.001	0.78 (0.68–0.89)	−0.38 (0.07)	<0.001	0.68 (0.59–0.78)	−0.23 (0.09)	0.015	0.85 (0.73–0.99)	−0.32 (0.07)	<0.001	0.72 (0.63–0.83)
Structural support	−0.76 (0.20)	<0.001	0.50 (0.34–0.74)	−0.02 (0.11)	0.84	0.99 (0.81–1.21)	−0.08 (0.14)	0.56	0.91 (0.70–1.18)	−0.38 (0.16)	0.016	0.68 (0.50–0.93)
Received support	−0.54 (0.07)	<0.001	0.60 (0.52–0.70)	−0.30 (0.08)	<0.001	0.78 (0.67–0.90)	−0.43 (0.09)	<0.001	0.69 (0.58–0.82)	−0.53 (0.08)	<0.001	0.59 (0.51–0.68)
Provided support x Received support	−0.26 (0.06)	<0.001	0.80 (0.71–0.91)	−0.24 (0.05)	<0.001	0.80 (0.72–0.90)	NS	–	–	−0.29 (0.06)	<0.001	0.74 (0.66–0.83)
Provided support x Structural support	NS	–	–	NS	–		NS	–	–	NS	–	–

*B* unstandardized coefficient, *SE* standard error, *OR* odd ratio, *95%CI* 95% confidence interval, *NS* not significant (*p* > 0.0125).

Coefficients and odds ratios are adjusted for age, gender, race/ethnicity, education, marital/partnered status, occupational status, income, enlistment status, combat veteran status, years of military service, number of medical conditions, adverse childhood experiences, direct and indirect trauma exposures, and frequency of social engagement with friends and family. Analyses of suicidal ideation are additionally adjusted for lifetime major depressive, posttraumatic stress, alcohol and drug use disorders.

Measures of provided, structural, and received support were standardized and evaluated as main effects in Step 1 of separate hierarchical logistic regression models; interaction terms were then incorporated in Step 2 of these models.

**Fig. 2:**
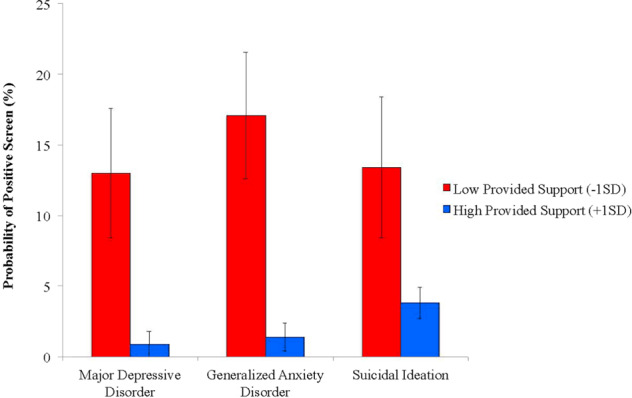
Probabilities of positive screen for major depressive disorder, generalized anxiety disorder, and suicidal ideation among veterans who reported high received social support (+1 SD) as a function of low (−1SD) and high (+1 SD) provided support. Red Bar—Low Provided Support (−1 SD), Blue Bar—High Provided Support (+1 SD). SD = standard deviation for the full sample. Error bars are 95% confidence intervals based on weighted and fully adjusted regression models. Probabilities are adjusted for age, gender, race/ethnicity, education, marital/partnered status, occupational status, income, enlistment status, combat veteran status, years of military service, number of medical conditions, adverse childhood experiences, direct and indirect trauma exposures, and frequency of social engagement with friends and family. Analysis of suicidal ideation is additionally adjusted for lifetime major depressive, posttraumatic stress, alcohol and drug use disorder.

For the interaction of received and provided support in predicting MDD, among veterans who had a 1 SD or greater than the mean score for received support, those who also had a 1 SD greater than the mean score for provided support had a 14-fold lower probability of screening positive for MDD than those who had a 1 SD lower than mean score for provided support (0.9% vs. 13.0%; see Fig. 2).

For the interaction of received and provided support in predicting GAD, among veterans who had a 1 SD or greater than the mean score for received support, those who also had a 1 SD greater than the mean score for provided support had a 12-fold lower probability of screening positive for GAD than those who had a 1 SD lower than mean score for provided support (1.4% vs. 17.1%; see Fig. 2).

For the interaction of received and provided support in predicting SI, among veterans who had a 1 SD or greater than the mean score for received support, those who also had a 1 SD greater than the mean score for provided support had a 3.5-fold lower probability of screening positive for SI than those who had a 1 SD lower than mean score for provided support (3.8% vs. 13.4%; see Fig. 2).

## Discussion

Results of this nationally representative study revealed that the majority of the US veteran population (60–72%) reported frequent engagement in providing various kinds of social support to others. Personality factors, notably agreeableness, conscientiousness, and openness to experiences, as well as greater received social support were strongly associated with the frequency of provided support. Provision of social support was independently associated with internalizing mental disorders (i.e., MDD, GAD, PTSD), as well as SI, with each standard deviation increase in provided support associated with a 22–32% reduced odds of these outcomes. Significant interaction effects were observed between provided and received/structural support, with veterans who reported high received and provided support having 3.5- to 14-fold reduced probability of screening positive for MDD, GAD, and SI relative to veterans who reported high received social support but the low provision of social support.

Higher income was associated with greater provision of social support. One interpretation of this finding is that greater income or socioeconomic status may increase the likelihood of engaging in the provision of social support, as greater resources have been linked to larger social networks^27,28^. Further, being married/partnered was associated with a higher level of provision of social support. This finding aligns with previous literature suggesting that married individuals are more likely to volunteer or provide social support than unmarried individuals^29^.

The high prevalence of provided social support among veterans is noteworthy and encouraging. While veterans are often identified as a high-risk population for adverse mental health outcomes, accumulating evidence has demonstrated that military experience may also be linked to increased resilience and post-traumatic growth^30–32^. For example, a study of 1287 male veterans aged between 44 to 91 years (40% combat veterans) found that perceiving benefit from stressful military experiences mitigated the negative consequences of combat exposure^32^. In the present study, positive perceptions of military experience were independently linked to higher scores on a measure of provided support. This finding extends prior work from our group showing that positive perceptions of military service are linked to reduced likelihood of mental disorders and suicidal thinking in veterans^33^. One interpretation of this finding is that veterans who perceived greater benefits from their military experience, which include factors such as cooperation and teamwork, dependability, and lifelong friendships^32^, may be less likely to develop mental health problems, which may in turn increase their engagement in altruistic behaviors. Alternatively, positive perceptions of military service may lead to greater engagement in altruistic behaviors, which may in turn mitigate risk for mental health problems. Further, while there is a large literature on prosocial behavior and altruism, which represent broad constructs, this study focused specifically on the provision and receipt of social support. Additional research is needed to determine whether the findings are specific to social support or prosocial behaviors more broadly^34^, as well as to disentangle longitudinal associations among these variables.

The positive association between the Big-Five personality factors, particularly agreeableness, conscientiousness, and emotional stability, and provision of social support accords with extant literature. Given that agreeableness is positively associated with age (i.e., as one age, they are more likely to become more agreeable)^35^ and that the majority of our sample was comprised of older veterans, the positive association between personality factors and provision of social support may, at least in part, be driven by older age. Previous studies have similarly identified associations between personality factors such as extraversion and agreeableness and received social support, as well as provided support^36–38^. One plausible explanation for these findings is that those who scored highly on both received and provided support are highly socially connected, and thus this association is bidirectional and mutually sustained. Personality factors and received support may also interact. For example, a previous longitudinal study of patients with chronic kidney disease found that greater receipt of social support among individuals with greater agreeableness was associated with a decrease in depressive symptoms, whereas support had little effect on depression change for individuals who scored lower in agreeableness^39^. Collectively, these findings underscore the importance of trait personality characteristics as potential drivers of the provision of support, as well as moderators of the protective effects of received social support.

The robust independent associations between the provision of social support and various adverse mental health outcomes observed in this study add to the existing literature on the protective effects of volunteering/civic engagement on adverse mental health outcomes^14,15^. Further, the findings of significant interactions between provided and received social support in relation to certain adverse mental health outcomes highlight the importance of considering the multi-faceted nature of social connectedness when examining associations with mental health outcomes. Our results revealed substantially reduced probabilities of MDD, GAD, and SI for veterans who reported both greater provided and received support relative to greater received support alone. Several neurobiological mechanisms may underlie these associations. For example, subcortical neural regions critical in parental care, such as greater activity in the ventral striatum and septal area^40^, and reduced amygdala activity^41^ have been linked to greater provision of support behaviors. The reinforcing-related neural mechanisms in the ventral striatum and septal area related to providing support have also been proposed to promote emotional and social well-being^11^. Conversely, greater amygdala activity to acute stressors has previously been linked to greater elevation in blood pressure^42^ and proinflammatory cytokines^43^. Taken together, these findings implicate fear and reward processing as possible neural mechanisms that mediate the relation between the provision of social support and reduced stress and risk for adverse mental health outcomes. Lastly, it is also plausible that veterans who could provide greater social support may have more access to other resources outside the VA system.

The results of this study have several clinical implications. In the United Kingdom, there have been recent increasing efforts to promote social prescribing as a means of helping improve both the mental and physical health of patients who present to their primary care physicians^44^. In the US, the VA offers a tele-support program called Compassionate Contact Corps to veterans who are socially isolated to talk regularly with trained volunteers via phone or video calls^45^. In addition, previous research has demonstrated that peer-outreach interventions may help improve depressive symptoms, as well as loneliness^46,47^. Volunteerism, which encourages physical activity, social connection, and meaningful purpose, has also been linked to improved mental well-being, social capital, and personal empowerment^48,49^. Results of our study suggest that interventions and social prescriptions to promote the ‘provision of support to others’ may be another avenue to potentially mitigate the risk for adverse mental health outcomes in veterans. The VA may be one setting in which interventions to bolster the provision of social support could be potentially implemented. For example, policies that expand the current peer specialist system within the VA mental health services^50^, as well as the VA’s Justice Programs^51^, may be considered. However, it should also be noted that nearly half of veterans do not utilize the VA for medical services^52^, so implementation and evaluation of initiatives outside of the VA system are also needed.

In the future, it may be helpful to develop manualized training to provide social support to others and train individuals who have psychiatric disorders or are at high risk of adverse mental health conditions. Alternatively, clinicians could assign tasks to provide social support via clinical interventions that are designed to promote certain behaviors, such as behavioral activation^53,54^. However, given the cross-sectional nature of the study, it is also possible that veterans with MDD, GAD, and SI reported substantially lower provided support. Longitudinal studies are needed to disentangle the temporal/causal nature of these associations, and consider the role of provided support, as well as other aspects of social support (e.g., structural, received support) in relation to mental health outcomes. Given that individuals may have different “profiles” of social connectedness (e.g., high received support, low provided support), it is also be helpful to evaluate personalized approaches to assessing, monitoring, and bolstering domains of social connectedness.

This study has four notable limitations. First, while nationally representative, the sample consisted primarily of older, male, and White, non-Hispanic veterans. Thus, it is unclear whether the results of the study may generalize to younger, more diverse samples of veterans or non-veteran population. Second, as noted above, the cross-sectional design does not make it possible to ascertain temporal/causal associations between the provision of social support, other aspects of social support (e.g., structural support, received support) and adverse mental health outcomes. The provision and receipt of social support can also be time- and context-specific, which may further limit the interpretation of causality in this study. Third, the measures utilized in this study to assess social support do not reflect the possibility that close social relationships with friends or family members may also be non-supportive, or potentially even detrimental, especially during stressful times^55^. Fourth, screening instruments were utilized to assess mental disorders. Further research utilizing structured clinical interviews is needed to replicate the results reported herein.

Notwithstanding these limitations, the results of this study provide the first-known population-based data on the prevalence and correlates of providing social support to US military veterans. They suggest that the majority of veterans frequently engage in some form of providing support and that greater engagement in such activities is linked to a significant reduction in the odds of internalizing psychopathology, as well as SI. Additional research is needed to replicate and extend these results in a prospective cohort, and in more diverse veteran and non-veteran populations; elucidate the mechanism of interaction between provided, received, and other aspects of social support, and their independent and interactive effects on adverse mental health outcomes; and evaluate the efficacy of interventions designed to bolster engagement in the provision of social support to others in mitigating risk for adverse mental health outcomes in veterans and other at-risk populations.

## Data Availability

Data are available from the senior author upon reasonable and explicit request.
